# Preliminary Effects of Fertilization on Ecochemical Soil Condition in Mature Spruce Stands Experiencing Dieback in the Beskid Śląski and Żywiecki Mountains, Poland

**DOI:** 10.1007/s11270-014-1971-4

**Published:** 2014-05-15

**Authors:** Stanisław Małek, Kazimierz Januszek, William S. Keeton, Józef Barszcz, Marek Kroczek, Ewa Błońska, Tomasz Wanic

**Affiliations:** 1 Department of Forest Ecology, Faculty of Forestry, University of Agriculture in Krakow, Al. 29-go Listopada 46, 31-425 Kraków, Poland; 2 Department of Forest Soil, Faculty of Forestry, University of Agriculture in Krakow, Al. 29-go Listopada 46, 31-425 Kraków, Poland; 3 Rubenstein School of Environment and Natural Resources University of Vermont, Burlington, VT 05405 USA

**Keywords:** Ecochemical indicators, Slow-release fertilizers, Old *Picea abies* stands, Poland

## Abstract

In recent years, there has been the phenomena of spruce dieback in Europe. Significant areas of spruce low mortality now cover both sides of the Polish southern border. We evaluated ecochemical parameters influencing the heavy dieback occurring in mature spruce stands in the Polish Carpathian Mountains. Dolomite, magnesite and serpentinite fertilizers were applied to experimental plots located in 100-year-old stands in the autumn of 2008. The experimental plots were located in the mid-elevational forest zone (900–950 m) on two nappes of the flysch Carpathians: Magura (Ujsoły Forest District) and Silesian (Wisła Forest District). The saturation of the studied soils demonstrates moderate resilience of soils in Wisła Forest District in relation to acid load and high flexibility of the Ujsoły soils. After application of the fertilizers, an increase of Mg, Ca and Mb was noted in the soil solution, determined in the overlaying highly acidic organic horizons through the ion-exchange buffering mechanism of highly protonated functional groups with high buffering capacity. Magnesium concentration increased following fertilization, presenting a potential improvement of forest growth capacity without the hazard of adverse side effects of liming. Aluminium stress in old spruce is unlikely, while trees in the control plots in Wisła Forest District may already be sensitive to aluminium stress. Serpentinite fertilization improved the supply of soils in magnesium without causing significant changes in the pH of the soil. Such changes in the pH were found in dolomite and magnesite fertilizer.

## Introduction

Polish spruce (*Picea abies*) forests, especially in western part of the Beskidy Mountains, have experienced significant dieback in recent years (Małek et al. 2012a, b). Previous studies indicate that sustainability of the forest in this area is particularly at risk in high and medium altitude locations because of the dominance of pure spruce stands and additional pressures related to abiotic, biotic and anthropogenic factors, particularly air pollution. The latter combined with the long-term effects of the natural acidification of soils by spruce have contributed to increase acidification of soils (Staszewski et al. 1999; Bytnerowicz et al. 1999; Małek 2010; Šrámek et al. 2010).

Both sulphur and nitrogen in the form of NH_4_^+^ contribute to soil acidification, but the S deposition rate has decreased in the last two decades, whereas the deposition of N seems to be constant or slightly increasing (Małek et al. 2012a, b; Šrámek et al. 2010). Therefore, the role of N in forest dieback has become an issue of growing concern (e.g. Van Breemen and Van Dijk 1987; Aber 1992; Hornung and Sutton 1995; Flower et al. 2007; Sicard et al. 2007; Małek 2010).

Together with the high nitrogen deposition (Małek 2010) and nutrient loss caused by canopy leaching of K, Ca, Mn and Mg (Ulrich 1983; Bredemeier 1988; Draaijers and Erisman 1995; Draaijers et al. 1997; Małek 2010; Šrámek et al. 2010), the following properties of precipitation, throughfall and soil solution have become modified: acid-neutralizing capacity (Reuss and Johnson 1986; Heinrichs et al. 1994; Jóźwiak and Kozłowski 2004; Małek 2009; Małek et al. 2012a, b), alkalinity (Harriman et al. 1990; Block et al. 2000; Jóźwiak and Kozłowski 2004; Małek 2009), soil acidity and base cation saturation (Ulrich 1983; Kowalkowski 2002) following soil acidification (Falkengren-Grerup et al. 1987), as well as Ca:Al ratio (Cronan and Grigal 1995) and BC:Al ratio (Sverdrup and Warfvinge 1993). These processes may increase tree demand for mineral nutrients, cause nutrient deficiency in the trees and change relations between elements (Cape et al. 1990; Zwoliński 2003). The properties listed above can be good ecochemical indicators of forest soil conditions and stand damage from acidification (Block et al. 2000; Kowalkowski 2002).

We hypothesized that (1) fertilization can be used to reduce susceptibility to forest dieback and (2) fertilization improves the chemical properties of soil and soil solution.

## Materials and Methods

Because of site differences related to surficial geology, the experimental plots were set up in the areas of two Carpathian nappes, differing in terms of lithological deposits. The Magura Nappe (Ujsoły Forest District) is built of thin-bedded sandstone with a share clay-marley slate inclusions, producing clay dominate waste-mantle with meso/eutrophic soils that is more buffered and resistant to degradation. The Silesian Nappe (Wisła Forest District) in the range of Barania Góra mountain, built of the lower Istebna layers, consists of thick-bedded sandstones and conglomerates, producing sandy-loam, gravelly-cobbly waste-mantle with oligotrophic soils. These are comparatively more susceptible to degradation (Małek et al. 2010).

The Haplic Podzol soil built of very stony, coarse sandy loam stratiform of very cobbly sandy loam with mor humus was noted on the experimental plots in Wisła. The Endoeutric Cambisol built of loam deposition on very cobbly clay loam and very cobbly silty clay loam with the moder type of humus was noted on the experimental plots in Ujsoły. A more comprehensive description of the soils in the Wisła and Ujsoły research plots is presented by Januszek et al. (2011).

Soil samples were collected on the experimental sites in Wisła on the 14th and in Ujsoły on the 16th of September 2010 from five plots of different nutrition treatment variants (dolomite—D, magnesite—M and serpentinite—S), as well as from reference plots where no nutrition treatment had been applied (control—C; Table 1). On the experimental sites in Wisła, from every plot (10 × 10m each), three aggregate samples were collected from four holes randomly placed within a plot with the size of 20 × 20 × 35 cm. The first sample was obtained from the organic horizon (without further subhorizons), one was taken from the AE horizon down to 20 cm and a third from 20 to 35 cm in depth. The sample taken from 20–35 cm depth came from the B and AE horizons, more seldom from the AE horizon because of the different thickness of the AE horizon. The soil samples on the experimental sites in Ujsoły were obtained from the depth of 0–10, 10–20 and 20–35 cm of the same research plots as in Wisła. The samples were marked by symbols OhA, AB and B regardless of the actual thickness of the OhA, AB and B horizons. The soil for determining chemical and physical properties was collected after removal of the litter layer. In all the cases, samples for the research were collected from 4 sub-stands of the soil horizon. Samples were returned to the laboratory for analysis.

**Table 1:** Content of macro- and some microelements in fertilizer and pH of fertilizer

Fertilizer	pH in H_2_O	pH in KCl	C	N	S	Na	K	Ca	Mg	Fe	Mn	Zn	Cu	Cd	Ni	Pb	Cr
			%								mg kg^−1^ of soil						
Dolomite	8.55	8.58	11.43	0.003	0.029	0.017	0.06	18.78	9.49	1.30	1,244.2	76.9	2.1	0.55	2.35	30.86	48.48
Magnesite	8.45	8.43	11.47	0.007	0.005	0.007	0.003	0.28	25.89	0.93	359.6	7.0	2.3	0.30	304.07	0.45	6.38
Serpentinite	8.66	8.38	1.67	0.001	0.011	0.009	0.006	0.39	18.10	5.77	621.5	38.4	11.5	1.96	1,673.21	1.25	131.5

The samples collected were first dried at room temperature and then sieved through a 2-mm sieve. The sample parameters and method of analysis are presented in Table 2.

**Table 2:** The sample parameters and method of analysis

Analyzed parameters	Methods
Soil pH	Determined potentiometrically, in H_2_O and 1 M KCl dm^−3^ solutions, with soil-to-solvent proportion of 1:2.5 for mineral soils and 1:5 for organic soils
Total C (C_T_) and total N (N_T_)	CNS 2000 Leco elemental analyzer with the calculation of the C:N ratio (in two horizons)
Hydrolytic acidity (total acidity—Hh)	Kappen method in the extract of 1 M of calcium acetate
Exchangeable acidity (Hw), exchangeable aluminium (H_Al_), exchangeable hydrogen (H_H_)	Sokołow method
Calcium (Ca^2+^), magnesium (Mg^2+^), potassium (K^+^) and sodium (Na^+^) (base exchange capacity—S)	In a 1-M CH_3_COONH_4_ extract of pH 7.0 as determined with a Thermo Scientific iCAP 6000 ICP OES Spectrometer, with calculation of effective cation exchange capacity T_e_ (total of S and H_w_) and the effective base saturation V_e_% (S/T_e_∙100)
Available phosphorus	Bray-Kurtz method
Content of Cr and Ni	Soluble in 1 mol dm^−3^ extract of 1 M HCl solution

We computed the percentages of exchangeable calcium, magnesium, potassium, sodium, aluminium and hydrogen. These were calculated in units of T_e_, the molar proportions of the exchangeable calcium and magnesium forms, the proportion of the total of exchangeable calcium, magnesium and potassium to exchangeable aluminium and the proportion of exchangeable calcium to exchangeable aluminium.

Analysis of the chemical composition of soil water was performed using gravitational and non-isolated (L-20) soil lysimeters, placed at the depth of 20 cm, vertically and horizontally penetrating the surface soil layers. They were installed in three repetitions for each experimental variant before the application of the fertilizers in September 2008. The surface area of each lysimeter was 0.077 m^2^. It was connected by means of a plastic tube to the collection container (a chemically neutral plastic container) placed in mineral soil (Małek 2009).

Sampling was performed at the beginning and the end of the growing season (August) in 2010. Mean pH and conductivity were measured directly on the sampling spot by means of equipment made by Eijkelkamp.

Water samples were analyzed using ion chromatography (Dionex-320, Sunnyvale, CA, USA) in order to determine the concentration of the following ions: Cl^−^, NO_3_^−^, SO_4_^2−^_,_ PO_4_^3−^, F^−^, NH_4_^+^, Na^+^, K^+^, Ca^2+^, Mg^2+^ and Al^3+^. We used ICP OES technology in order to determine the elements Fe, Mn, Zn and Ni. Parallel analysis was performed for the reference material with the certified content of the analyses. For this purpose, we used a water sample with low pH from south Ontario (Canada), RAIN.97—no. 409.

The results obtained were used to calculate the following ecochemical soil indicators: ANC_aq_, ALK, Ma, Mb, BS, Ca/Al and BC/Al (Kowalkowski 2002; Małek 2009; Małek et al. 2012a, b). The acid-neutralizing capacity (ANC_aq_) (Reuss and Johnson 1986; Heinrichs et al. 1994), alkalinity (ALK) (Harriman et al. 1990; Block et al. 2000), the degree of soil acidity (Ma%) (Ulrich 1983), acidic cations (Ma), saturation of the exchangeable complex of the solid soil phase with alkalis (Mb), saturation with alkalis (BS) (Kowalkowski 2002) and molar ratios Ca:Al (Cronan and Grigal 1995) and BC:Al (Sverdrup and Warfvinge 1993).

Statistical data analysis was performed using Statistica 9 software. Differences between the mean values were evaluated with the nonparametric Kruskal-Wallis test. We also calculated Pearson’s correlation coefficients for the purpose of assessing the physico-chemical properties of soil and soil water.

## Results

A significant impact of fertilization variants on soil properties was found 2 years after treatment. Based on the comparison of three types of fertilization, we noted a more radical change in the properties of the surface levels of two plots after dolomite and magnesite fertilization and a less radical change in the properties of the surface levels after serpentinite fertilization. On the Wisła plot of podzolic soil (Table 3), after the higher dose of fertilization (4,000 kg/ha), we observed a more radical change in the properties of the surface levels. After fertilization with the lower dose of fertilizer (2,000 kg/ha), less pronounced changes in the levels of surface cambisol were noted on the Ujsoły plot (Table 4).

**Table 3:** Mean values (x), standard deviations (SD), statistics (H) and statistical probability (*p*) (from five replications) of soil properties in O, AE and B horizons of soil on control plots (C), 2 years after fertilization with dolomite (D), magnesite (M) and serpentinite (S) in Wisła Forest District on the basis of the Kruskal-Wallis test

Soil properties	C		D		M		S		H	*p* value
	x	SD	x	SD	x	SD	x	SD		
Ofh horizon										
pH in H_2_0	3.93^a^	0.09	5.59^b^	0.36	5.30^b^	0.50	4.25^ab^	0.12	16.49	0.0009
pH in KCl	2.88^a^	0.10	4.93^b^	0.45	4.35^b^	0.64	3.26^ab^	0.10	16.92	0.0007
Hh	87.47^a^	10.56	44.02^b^	11.32	48.30^b^	15.01	75.31^ab^	3.48	15.14	0.0017
H_H_	2.65^a^	1.21	0.39^b^	0.20	0.67^b^	0.43	1.55^ab^	0.62	14.25	0.0026
H_Al_	13.17^a^	1.63	0.95^b^	0.53	2.10^b^	1.48	8.43^ab^	1.39	16.55	0.0009
S	6.33^a^	1.08	39.10^b^	3.91	27.04^b^	6.06	14.04^ab^	2.28	17.58	0.0005
T_e_	22.15^a^	1.62	40.45^b^	4.13	29.81^ab^	4.50	24.03^a^	1.78	16.28	0.0010
Ve%	29.0^a^	7.0	96.7^b^	1.2	90.0^b^	7.6	58.3^ab^	7.5	16.71	0.0008
C_T_	33.9	4.8	29.5	4.1	27.9	5.1	32.2	3.6	5.95	0.1141
N_T_	1.342	0.138	1.152	0.171	1.094	0.230	1.287	0.172	5.38	0.1462
C:N	25.2	1.4	25.6	1.4	25.6	1.2	25.1	1.3	0.85	0.8371
Ca^2+^ [mg kg^−1^]	925.0^ab^	209.1	4,828.6^a^	544.2	848.9^ab^	117.8	882.5^b^	113.1	10.93	0.0121
Mg^2+^ [mg kg^−1^]	112.4^a^	12.1	1,744.9^bc^	144.5	2,686.6^b^	775.7	1,055.6^ac^	263.9	17.86	0.0005
K^+^ [mg kg^−1^]	282.2	45.5	229.0	39.9	256.8	36.6	320.8	89.8	7.69	0.0530
Na^+^ [mg kg^−1^]	16.2	3.0	17.2	7.3	12.8	3.7	14.4	3.3	2.12	0.5479
P av. [mg kg^−1^]	18.4	4.5	19.8	4.2	17.9	2.2	20.0	2.1	2.04	0.5641
Ca:Mg	5.03^a^	1.15	1.68^a^	0.07	0.21^b^	0.07	0.49^b^	0.10	17.86	0.0005
(Ca + Mg + K):Al	0.49^a^	0.15	47.18^b^	14.32	23.74^b^	22.62	1.73^ab^	0.58	16.55	0.0009
Ca:Al	0.36^a^	0.13	29.09^b^	8.87	3.20^ab^	2.43	0.52^a^	0.15	16.71	0.0008
Ni [mg kg^−1^]	5.15^a^	1.69	3.79^a^	1.23	12.70^ab^	5.64	25.77^b^	5.69	16.19	0.0010
Cr [mg kg^−1^]	3.00	2.31	4.05	3.37	4.60	3.54	2.89	0.98	0.58	0.9016
AE horizon										
pH in H_2_0	3.80^ab^	0.08	3.98^a^	0.15	3.83^ab^	0.11	3.71^b^	0.08	10.39	0.0156
pH in KCl	2.90	0.14	2.99	0.14	2.99	0.10	2.91	0.07	4.08	0.2523
Hh	21.10	4.20	17.87	4.90	19.64	5.99	17.51	3.96	2.16	0.5387
H_H_	0.39	0.19	0.49	0.27	0.48	0.10	0.54	0.30	1.09	0.7794
H_Al_	12.73	3.62	9.10	3.54	11.17	3.81	9.07	3.10	3.43	0.3294
S	0.54	0.18	1.28	0.77	1.27	0.78	0.61	0.20	5.78	0.1230
T_e_	13.66	3.72	10.86	4.05	12.92	4.37	10.21	3.03	2.13	0.5456
Ve%	4.2^a^	1.8	11.1^b^	2.9	9.4^ab^	4.7	6.0^ab^	1.2	10.77	0.0130
C_T_	5.08	0.78	4.69	1.75	5.09	2.12	5.02	1.58	0.34	0.9529
N_T_	0.258	0.033	0.230	0.086	0.265	0.125	0.251	0.071	0.60	0.8964
C:N	19.7	1.6	20.4	0.9	19.6	1.8	19.9	0.9	3.11	0.3753
Ca^2+^ [mg kg^−1^]	38.3^ab^	23.7	96.8^a^	62.7	27.8^b^	11.9	29.6^ab^	9.8	9.35	0.0249
Mg^2+^ [mg kg^−1^]	20.3^a^	9.5	76.7^ab^	53.0	114.3^b^	77.9	39.4^ab^	20.8	9.99	0.0186
K^+^ [mg kg^−1^]	62.6	9.6	56.3	15.0	69.6	33.4	55.3	12.2	1.95	0.5831
Na^+^ [mg kg^−1^]	4.3	0.8	4.1	0.9	3.9	0.9	3.9	1.1	0.87	0.8316
P av. [mg kg^−1^]	10.7	8.4	8.7	5.9	4.7	2.5	8.2	3.8	2.07	0.5586
Ca:Mg	1.19^a^	0.63	0.81^a^	0.20	0.19^b^	0.08	0.48^ab^	0.22	12.91	0.0048
(Ca + Mg + K):Al	0.04^a^	0.02	0.13^b^	0.04	0.11^ab^	0.06	0.07^ab^	0.02	10.77	0.0130
Ca:Al	0.02^ab^	0.01	0.05^a^	0.02	0.01^b^	0.00	0.02^ab^	0.01	10.31	0.0161
Ni [mg kg^−1^]	1.37	0.74	3.10	3.86	2.14	1.30	2.44	1.54	1.36	0.7140
Cr [mg kg^−1^]	1.63	0.95	3.64	4.75	2.30	1.08	2.54	1.69	1.93	0.5880
B horizon										
pH in H_2_0	3.99	0.18	4.08	0.15	3.95	0.15	4.05	0.11	1.91	0.5913
pH in KCl	3.18	0.16	3.17	0.21	3.23	0.18	3.19	0.15	0.51	0.9149
Hh	20.59	4.66	18.50	3.26	19.23	4.30	19.57	4.28	1.21	0.7516
H_H_	0.28	0.26	0.36	0.21	0.39	0.45	0.31	0.28	0.66	0.8833
H_Al_	17.06	5.18	13.95	3.51	15.39	5.29	14.48	4.22	2.73	0.4359
S	0.31^a^	0.03	0.51^b^	0.13	0.35^ab^	0.06	0.33^ab^	0.04	11.73	0.0084
T_e_	17.64	5.09	14.81	3.24	16.14	5.08	15.12	2.29	2.20	0.5319
Ve%	1.93	0.91	3.76	2.02	2.31	0.64	2.29	0.57	6.95	0.0734
Ca^2+^ [mg kg^−1^]	19.2^ab^	7.4	38.4^a^	18.5	15.9^b^	5.7	19.2^ab^	6.8	8.62	0.0348
Mg^2+^ [mg kg^−1^]	9.5^a^	1.5	23.9^b^	6.2	20.0^b^	3.8	13.7^ab^	3.1	14.66	0.0021
K^+^ [mg kg^−1^]	43.8	7.9	40.3	6.7	36.4	5.8	40.5	1.6	2.75	0.4320
Na^+^ [mg kg^−1^]	4.5	2.0	4.2	1.0	3.7	0.2	4.8	1.3	1.42	0.7002
P av. [mg kg^−1^]	1.26	0.56	0.96	0.29	0.49	0.17	0.93	0.41	7.21	0.0656
(Ca + Mg + K):Al	0.02	0.01	0.04	0.02	0.02	0.01	0.02	0.01	6.63	0.0845
Ca:Al	0.01	0.01	0.02	0.01	0.01	0.00	0.01	0.00	6.27	0.0993
Ni [mg kg^−1^]	0.68	0.71	0.78	0.58	0.58	0.49	0.92	0.51	0.96	0.8101
Cr [mg kg^−1^]	2.56	0.34	2.12	0.35	2.05	0.50	2.76	0.93	4.62	0.2016

Different small letters in the upper index of the mean values mean significant differences. Explanation for Table 3, see Materials and Methods

**Table 4:** Mean values (x), standard deviations (SD), statistics (H) and statistical probability (*p*) (from five replications) of soil properties in OhA, AB and B horizons of soil on control plots (C), 2 years after fertilization with dolomite (D), magnesite (M) and serpentinite (S) in Ujsoły Forest District on the basis of the Kruskal-Wallis test

Soil properties	C		D		M		S		H	*p* value
	x	SD	x	SD	x	SD	x	SD		
OhA horizon (0–10 cm)										
pH in H_2_0	4.28	0.21	4.72	0.34	4.68	0.32	4.54	0.27	5.58	0.1337
pH in KCl	3.40	0.23	3.69	0.32	3.62	0.21	3.53	0.21	3.35	0.1314
Hh	35.53	6.46	24.93	8.26	29.96	5.66	30.55	5.91	3.57	0.3116
H_H_	0.32	0.15	0.20	0.13	0.16	0.03	0.15	0.08	7.16	0.0669
H_Al_	10.30	4.60	6.95	3.56	7.07	1.40	9.20	3.44	3.54	0.3160
S	7.59	3.93	13.78	5.66	11.23	3.98	9.82	1.88	3.87	0.2760
T_e_	18.21	1.42	20.93	4.68	18.47	4.68	19.17	3.09	1.97	0.5784
Ve%	42.2	23.3	64.3	19.4	59.9	7.8	52.0	10.9	4.92	0.1778
C_T_	12.25	4.08	10.84	5.51	11.25	2.68	11.99	3.17	0.78	0.8536
N_T_	0.58	0.13	0.55	0.21	0.55	0.10	0.55	0.08	0.55	0.9068
C:N	20.6	2.7	18.9	2.5	20.1	1.9	21.4	3.3	3.27	0.3512
Ca^2+^ [mg kg^−1^]	1,233.3	715.8	2,084.2	1,001.9	1,100.3	486.5	1,342.8	475.0	4.33	0.2284
Mg^2+^ [mg kg^−1^]	105.6^a^	38.6	351.1^ab^	151.5	621.1^b^	178.4	319.8^ab^	92.1	14.86	0.0019
K^+^ [mg kg^−1^]	197.5	48.6	167.8	36.5	222.4	122.9	165.5	47.6	2.53	0.4696
Na^+^ [mg kg^−1^]	14.9	3.6	14.7	2.2	15.5	5.7	14.4	4.5	0.26	0.9679
P av. [mg kg^−1^]	2.86	1.40	2.56	1.29	3.41	1.28	3.17	1.72	1.85	0.6041
Ca:Mg	6.75^a^	1.32	3.98^ab^	2.67	1.07^b^	0.29	2.89^ab^	1.70	13.78	0.0032
(Ca + Mg + K):Al	1.45	2.10	2.88	2.38	1.59	0.47	1.19	0.52	4.51	0.2115
Ca:Al	1.22	1.84	2.15	1.75	0.79	0.30	0.84	0.48	3.11	0.3743
Ni [mg kg^−1^]	6.10	2.53	6.10	3.34	5.00	0.67	9.53	2.21	7.33	0.0621
Cr [mg kg^−1^]	3.88	2.15	3.32	1.90	1.94	0.93	1.66	1.05	4.71	0.1940
AB horizon (10–20 cm)										
pH in H_2_0	4.68	0.17	4.74	0.23	4.61	0.24	4.76	0.24	1.28	0.7330
pH in KCl	3.60	0.11	3.65	0.13	3.55	0.16	3.67	0.22	0.84	0.8378
Hh	16.56	2.47	15.30	1.70	16.39	2.89	15.11	3.25	0.61	0.8938
H_H_	0.21	0.09	0.32	0.09	0.28	0.09	0.28	0.03	3.87	0.2762
H_Al_	11.79	4.11	10.74	4.08	11.19	3.03	10.85	5.40	0.05	0.9969
S	4.03	1.15	6.55	4.91	3.87	1.33	6.13	4.28	1.38	0.7109
T_e_	16.03	3.21	17.60	1.74	15.34	2.47	17.26	1.96	4.01	0.2608
Ve%	26.7	11.9	36.2	25.6	26.0	10.7	36.7	28.0	0.28	0.9637
C_T_	3.02	0.58	2.77	0.55	2.93	0.51	3.09	0.54	0.58	0.9016
N_T_	0.23	0.03	0.23	0.04	0.22	0.02	0.23	0.04	0.87	0.8316
C:N	13.0	1.3	12.3	0.9	13.2	1.5	13.3	1.1	2.57	0.4635
Ca^2+^ [mg kg^−1^]	649.0	216.3	1,082.7	882.6	575.6	247.4	1,020.8	800.0	2.41	0.4926
Mg^2+^ [mg kg^−1^]	57.0	8.3	96.3	48.2	85.8	8.7	89.7	28.4	5.79	0.1224
K^+^ [mg kg^−1^]	112.0	20.4	121.8	48.1	100.1	17.5	101.2	27.2	1.48	0.6869
Na^+^ [mg kg^−1^]	8.1	1.7	9.7	1.1	9.3	1.0	9.9	1.8	3.26	0.3528
P av. [mg kg^−1^]	0.80	0.24	0.89	0.21	0.93	0.18	0.94	0.24	1.08	0.7810
Ca:Mg	6.82	1.65	6.04	2.06	4.00	1.36	6.34	2.64	6.33	0.0968
(Ca + Mg + K):Al	0.40	0.26	1.04	1.48	0.38	0.24	1.69	3.04	0.37	0.9461
Ca:Al	0.33	0.23	0.88	1.30	0.29	0.21	1.48	2.72	0.81	0.8481
Ni [mg kg^−1^]	3.85	0.93	4.74	2.40	3.75	0.98	5.16	2.43	2.63	0.4515
Cr [mg kg^−1^]	3.00	1.58	3.10	1.55	1.82	0.90	1.91	0.81	3.32	0.3449
B horizon (20–35 cm)										
pH in H_2_0	4.92	0.10	4.91	0.27	4.86	0.35	4.97	0.34	0.17	0.9816
pH in KCl	3.70	0.09	3.71	0.21	3.69	0.21	3.79	0.32	0.59	0.8992
Hh	12.57	1.74	12.94	2.87	12.16	3.47	11.71	3.40	0.65	0.8859
H_H_	0.27	0.05	0.20	0.05	0.18	0.11	0.20	0.06	4.35	0.2275
H_Al_	7.88	4.56	8.82	4.32	8.76	4.18	8.45	5.26	0.28	0.9637
S	5.57	0.69	7.38	5.45	5.86	2.55	7.32	3.57	0.37	0.9461
T_e_	13.72	5.00	16.39	1.48	14.80	1.89	15.98	1.98	2.08	0.5548
Ve%	45.6	19.1	43.4	28.9	41.3	20.3	48.2	28.8	0.39	0.9414
Ca^2+^ [mg kg^−1^]	1,110.3	927.02	1,254.5	998.1	965.9	468.4	1,248.9	675.1	0.39	0.9414
Mg^2+^ [mg kg^−1^]	78.1	15.7	96.9	47.7	91.9	22.6	97.6	21.4	1.79	0.6174
K^+^ [mg kg^−1^]	103.6	18.5	108.1	36.0	93.1	24.7	95.1	13.6	0.83	0.8418
Na^+^ [mg kg^−1^]	9.2	1.4	9.8	1.7	10.0	1.4	10.6	2.2	1.66	0.6452
P av. [mg kg^−1^]	0.58	0.12	0.65	0.17	0.72	0.17	0.71	0.18	2.03	0.5667
Ca:Mg	7.30	0.69	7.12	2.14	6.08	1.75	7.42	2.33	1.95	0.5831
(Ca + Mg + K):Al	1.34	1.60	2.69	4.90	1.00	0.66	6.87	13.96	0.44	0.9319
Ca:Al	1.11	1.32	2.37	4.38	0.83	0.59	6.14	12.56	0.57	0.9042
Ni [mg kg^−1^]	3.11	1.37	2.76	1.47	2.18	1.49	1.76	0.74	4.21	0.2395
Cr [mg kg^−1^]	4.37	0.36	4.11	1.94	3.56	0.78	3.96	1.17	3.40	0.3340

Different small letters in the upper index of the mean values mean significant differences. Explanation for Table 4, see Materials and Methods

In the Ofh horizon on the dolomite-fertilized plots, there noted a significant difference (*p* < 0,05) compared to the control plots on the Wisła plot. This held for a large majority of soil properties, apart from concentration, including total C and N, exchangeable sodium, available phosphorus, chromium soluble and C:N ratio (Table 3).

The results showed a higher average pH in H_2_O and KCl in the case of dolomite fertilization in Ofh horizon of Wisła plots, 1.66 and 2.05, respectively. For this treatment, hydrolytic acidity was less than half of control and had a lower concentration of hydrogen and exchangeable aluminium (Table 3). In the case of dolomite fertilization compared to the control, the AE horizon showed significantly higher values in pH in H_2_O (an average of 0.18 pH units), content of exchangeable calcium and magnesium form (2.5- and 3.8-fold on average) and a higher base saturation (6.9 % on average). The B horizon showed a significantly higher content of the exchangeable forms of calcium and magnesium (2 and 2.5 times higher, respectively) and a higher total base (an average of 0.2 cmol (+) kg^−1^; Table 3) compared to the controls.

**Fig. 1:**
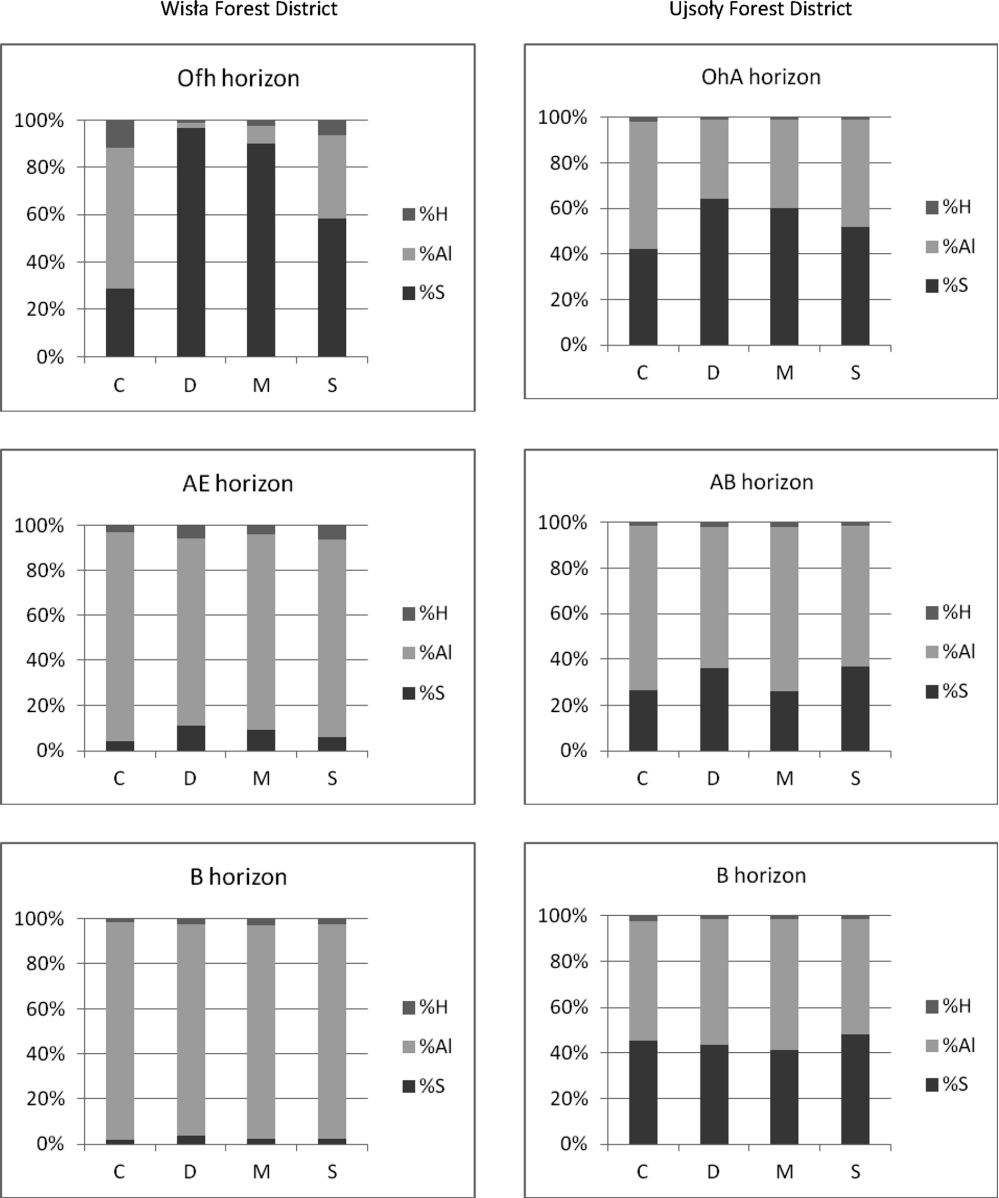
The share of base cations and share exchangeable Al and H in the sorption complex (%) in the surface levels of podzolic soil (Wisła Forest District) as well as brown cambisol, leached soil (Ujsoły Forest District) depending on the fertilization variant: *C* control; *D* dolomite; *M* magnesite; *S* serpentinite, in a dose of, respectively, 4 and 2 t ha^−1^ of the plot in Wisła and Ujsoły, 2 years after fertilization

Statistically significant changes in a great majority of the studied properties of the soil, in addition to the effective sorption capacity, the content of alkaline cation exchange, concentration of C and N, total exchangeable sodium, available phosphorus, nickel and chromium, the percentage of exchangeable forms of calcium, potassium and sodium in the capacity of the absorbing complex and the C:N and Ca:Al (Table 3), were reported in the Ofh horizon of Wisła experimental plot, magnesite-fertilized plots compared to control plots, as in the case of the dolomite-fertilized plots. A higher average pH in H_2_O and KCl (respectively, 1.37 and 1.47 pH units), a lower hydrolytic acidity (1.8-fold), a lower content of hydrogen and exchangeable aluminium (75 and 84 %), a higher value of the sum of bases (20.7 cmol (+) kg^−1^ on average) and a smaller share of aluminium and hydrogen (51.6 and 9.3 %; Fig. 1) were noted. A significantly lower ratio of exchangeable forms of calcium to magnesium (from 5.03 to 0.21) and a higher value of the sum of exchangeable calcium, magnesium and potassium to aluminium (from 0.49 to 23.74) were noted. Lower values of the ratio of the exchangeable form Ca:Mg (from 1.19 to 0.19) and a larger share of exchangeable magnesium in the sorption complex were noted in the AE horizon. These values were 5.7 % on average (Fig. 1). A significantly higher content of exchangeable magnesium, averaging 2.1-fold, was noted in the B horizon (Table 3).

Significant enrichment of exchangeable Mg, lower values of the ratio of the exchangeable form Ca:Mg (on average from 5.03 to 0.49) and a higher, approximately 5-fold, concentration of soluble nickel in 1 mol HCl dm^−3^ (Table 3) were noted in the Ofh horizon of the plots fertilized with serpentine in comparison to the control plots.

Soil properties in AE horizon were not significantly different from the plots fertilized with serpentinite as compared to the control plots. We found a 2.2-fold higher content of exchangeable magnesium and a higher concentration of exchangeable hydrogen in the AE horizon of soil on the plots fertilized with serpentinite, dolomite and magnesite (Table 3). The marked properties in the samples from the B horizon of soil of the serpentinite-fertilized plots, as compared with the properties of the B horizon on the control plots, were not significantly varied but had a higher content of exchangeable magnesium, and part of removable Mg in the sorption complex was noted (Fig. 1).

The AE horizon of the soil of the dolomite-fertilized plots as compared to the soil of the serpentinite-fertilized plots had significantly higher values of pH in H_2_O (about 0.27 units). In the Ofh and AE horizons of the plots with magnesite and serpentinite fertilization, we noted a lower content of exchangeable calcium compared to the control plots. This indicates a more intense rate of calcium leaching from soils fertilized with magnesite and serpentine than in soils without fertilization.

On the Ujsoły plot 2 years after fertilization in the OhA horizon (0–10 cm), the magnesite-fertilized plots showed statistically significant changes in the properties relative to the control plots. These differences included a higher content of exchangeable magnesium (an average of 5.9-fold), a higher share of exchangeable magnesium in the sorption complex (on average by 22.7 %) and a lower ratio of exchangeable calcium to magnesium (from 6.75 to 1.07; Fig. 1).

Higher concentrations of exchangeable hydrogen (Tables 3 and 4) and higher shares of H^+^ in the sorption complex (Fig. 1) were reported on the Wisła plot in the AE and B horizons and on the Ujsoły plot in the AB horizon. This may reflect an increase in the concentration of exchangeable hydrogen in the soil solution than in the sorption complex in these horizons immediately after fertilization.

Sampling period strongly affected the concentrations of chemicals in soil water related to the periods of their sampling is connected with the chemical composition of the fertilizers used. After the winter period, on plots with dolomite concentration, levels increased for NO_3_^−^, NH_4_^+^, SO_4_^2−^ and Ca^2+^ (in both locations); while plots with magnesite and serpentinite in the Wisła Forest District, there was an increase of K^+^, Mg^2+^, Fe and Al. Similar relations also held after the vegetation period but with higher concentrations of the analytes. After that period, the soil waters on the research plots in Wisła showed, in comparison with the control, a considerable increase of the concentrations of NO_3_^−^ and SO_4_^2−^ as well as Ca^2+^ and Mg^2+^, while the waters in Ujsoły additionally showed an increase in NH_4_^+^. These changes signify an increase in the mineralization of the organic matter and the process of nitrification, which is indicated by enzymatic activity and the rate of mineralization of the organic nitrogen compounds net (Haynes & Swift 1988; Valeur et al. 2000; Valeur et al. 2002; Januszek et al. 2011).

Also here, there may occur an influence of a decrease in the non-specific sorption of sulphates due to a reduction of acidification (Marschner 1993). A larger amount of potassium in soil solutions and the related leaching and threat of potassium deficiency may be related to a reduction of the selective sorption of monocations for the sake of doubly charged cations due to the fertilization applied (Kim et al. 2003) as well as due to further removal of Ca^2+^ by Mg^2+^ and increased removal of Al^3+^ and H^+^ from the sorptive complex.

After winter 2009/2010, water penetrating the 20-cm layer of soil treated with serpentinite became acidified, especially in Wisła Forest District. The acidification deepened after the vegetation period. In Ujsoły Forest District, the soil water reaction also fell (starting from higher initial values than in Wisła), but to a smaller extent (Tables 5 and 6). The dominant process of buffering in Wisła Forest District was the dissolution and complexing of metal hydroxides (Fe and Al buffers), whereas in Ujsoły Forest District, it was the release of Al from the crystalline networks of aluminosilicates (ion buffer).

**Table 5:** Concentrations of ions and metals (in mg/l) in soil waters from lysimeters in spring and autumn 2010 on research plots fertilized all over their surface in spruce stands in Ujsoły and Wisła Forest Districts in autumn 2008

Fertilization variant	Cl^−^	NO_3_^−^	SO_4_^2−^	Na^+^	K^+^	Ca^2+^	Mg^2+^	Fe	Mn	Al^3+^	NH_4_^+^	Zn
Wisła												
Spring												
C	1.500	9.400	9.700	0.987	2.511	2.369	0.303	0.393	0.053	0.488	1.155	0.026
D	1.516	10.900	10.625	1.004	2.838	2.644	0.347	0.390	0.052	0.435	1.298	0.025
M	1.583	10.079	10.900	1.002	2.794	2.590	0.383	0.366	0.061	0.483	1.368	0.047
S	1.450	9.775	9.925	0.972	2.631	2.486	0.365	0.311	0.075	0.457	1.358	0.062
Autumn												
C	1.550	9.675	9.750	0.994	2.578	2.456	0.306	0.394	0.055	0.428	1.545	0.037
D	1.515	11.070	10.925	1.015	2.724	2.744	0.354	0.401	0.066	0.454	1.278	0.040
M	1.615	10.120	10.950	1.024	2.738	2.640	0.393	0.469	0.068	0.449	1.400	0.030
S	1.490	9.800	9.950	1.003	2.795	2.566	0.379	0.485	0.060	0.419	1.395	0.067
Ujsoły												
Spring												
C	1.806	12.550	9.365	1.061	4.088	4.437	1.510	0.325	0.042	0.433	1.280	0.004
D	1.978	12.904	9.642	1.093	4.880	5.610	1.873	0.388	0.048	0.481	1.388	0.002
M	1.985	12.755	10.039	1.069	4.721	5.399	1.989	0.367	0.047	0.455	1.470	0.005
S	1.848	12.808	9.385	1.062	4.442	5.240	1.620	0.316	0.041	0.420	1.350	0.004
Autumn												
C	1.804	12.760	9.459	1.052	4.073	4.950	1.586	0.328	0.043	0.412	1.305	0.003
D	1.937	12.983	10.033	1.056	4.753	5.701	1.903	0.324	0.055	0.496	1.488	0.003
M	1.942	12.877	10.113	1.045	4.771	5.450	1.965	0.321	0.054	0.467	1.495	0.004
S	1.842	12.867	9.667	1.038	4.469	5.252	1.627	0.327	0.024	0.431	1.317	0.002

*C* control plots, fertilization with *D* dolomite, *M* magnesite, *S* serpentinite

**Table 6:** Indices of the ecochemical soil condition in the light of the results of analyses of soil waters sampled in spring and autumn 2010 on research plots fertilized all over their surface in spruce stands in Ujsoły and Wisła Forest Districts

Fertilization variant	pH	BS	ANC_aq_	ALK	Ma	Mb	Ma%	Ca/Al	Mb/Al	BC/Al
			meq L^−1^	mmol L^−1^						
Wisła										
Spring										
C	3.63	40.7	−18.850	−18.921	0.026	0.179	59.3	3.3	9.9	7.5
D	4.41	75.6	−21.248	−21.328	0.024	0.197	24.4	4.1	12.2	9.5
M	4.22	69.3	−20.703	−20.783	0.026	0.195	30.7	3.6	10.9	8.5
S	4.11	64.8	−19.436	−19.513	0.024	0.187	35.2	3.7	11.0	8.5
Autumn										
C	3.65	42.5	−19.168	−19.242	0.024	0.183	57.5	3.9	11.5	8.8
D	4.44	76.2	−21.715	−21.798	0.025	0.197	23.8	4.1	11.7	9.1
M	4.28	71.4	−20.791	−20.873	0.026	0.197	28.6	4.0	11.8	9.1
S	4.12	65.8	−19.476	−19.555	0.025	0.195	34.2	4.1	12.5	9.7
Ujsoły										
Spring										
C	4.87	89.9	−21.419	−21.591	0.023	0.324	10.1	6.9	20.2	17.3
D	5.58	93.2	−21.939	−22.156	0.026	0.389	6.8	7.9	21.9	19.2
M	5.27	92.8	−22.194	−22.410	0.024	0.384	7.2	8.0	22.8	20.0
S	5.28	92.9	−21.638	−21.835	0.022	0.357	7.1	8.4	23.0	20.0
Autumn										
C	4.86	90.5	−21.692	−21.880	0.022	0.339	9.5	8.1	22.2	19.2
D	5.60	93.5	−22.408	−22.629	0.025	0.388	6.5	7.7	21.1	18.6
M	5.27	93.1	−22.389	−22.606	0.024	0.384	6.9	7.9	22.2	19.6
S	5.28	92.9	−21.978	−22.176	0.022	0.357	7.1	8.2	22.4	19.6

*C* control plots, fertilization with *D* dolomite, *M* magnesite, *S* serpentinite

The saturation of the analyzed waters with alkalis (BS) was very low especially in Wisła Forest District (Table 5). In the soil waters of Ujsoły Forest District, the level of BS was, however, three times higher than that in the waters of Wisła. The values obtained on the research plots in Wisła Forest District show moderate flexibility of water solutions (after their passage through the surface soil level) in relation to the acid load, whereas the results from the plots in Ujsoły Forest District indicate high flexibility of the solutions (Table 6).

The opposite tendencies for the degree of soil acidity, Ma% (according to Ulrich 1983), were determined via analysis of waters which pass through it. The values obtained for this feature of waters from Wisła indicate the first acidity class (very acid soils), as in the Dupniański Stream catchment (Małek 2009); the Ma% values obtained in Ujsoły indicate the third class (weakly acid soils). The fertilization applied did not basically change these values despite a considerable increase in the saturation of the solid soil phase with the alkalis of the exchangeable complex (Mb) and the preservation of the acidic cations (Ma) on the same level (Table 6).

The acid-neutralizing capacity (ANC_aq_) of the analyzed waters slightly decreased in both locations and fluctuated in areas with an older stand, where it increased to approximately −18 to −22 mmol L^−1^, which—according to the scale applied—still situated these waters near the “0” value (Table 6). According to Kowalkowski (2002), soil waters with such alkalinity (from −7 to −23 mmol L^−1^) respond with strong fluctuations of pH values even to the smallest changes in the composition of the solution caused by the inflow of NO_3_^−^ and SO_4_^2^ anions.

An increase in pH reaction and saturation with alkalis (BS) was observed with a simultaneous decrease of the degree of acidity (Ma%), especially on the plots with dolomite and mostly in Wisła Forest District in 2010. The phenomenon intensified in the vegetation period. In the light of analysis of waters from lysimeters, fertilization with magnesite increased the saturation of the exchangeable complex of the solid soil phase with alkalis (Mb), mainly in Wisła Forest District. In this forest district, soils undergo the process of dissolving and complexing of metal hydroxides (aluminium buffer), whereas in Ujsoły Forest District, there occurs a release of alkaline cations from silicates and exchangers. The saturation of soils water under spruce stands in Wisła Forest District with alkalis is moderate, similarly to soils under mature stands in Dupniański Stream catchment (Małek 2009), whereas it was high in Ujsoły Forest District (Table 6).

The acid-neutralizing capacity (ANC_aq_) and alkalinity of waters from soil lysimeters in older spruce stands grew in 2010 after the winter period as well as after the vegetation period. The values of these characteristics were close to the “0” value, indicating similar relations and possibilities of changes on these plots to those occurring in stands of the Dupniański stream catchment (Małek 2009) but slightly weaker and slower. Molar ratios in the water from lysimeters under the old spruce stands defined the Al stress as improbable, and the release of alkaline cations from fertilizers in 2010 further improved their values (Table 6).

Among the analyzed properties of solutions obtained from soils on research plots in Wisła, what correlated most with the properties of the surface horizons of the analyzed soils were (Tables 7 and 8) the acid-neutralizing capacity (ANC), alkalinity (ALK) and the sum of alkaline cations (Mb; Table 7). The ANC and ALK values of the solutions sampled in the lysimeters correlated negatively with the pH of soil in H_2_O and in KCl, with the sum of alkaline cations (BC), with the degree of saturation of the sorptive complex with alkaline cations (V%) and with the molar ratio of the sum of alkaline cations (BC) to the exchangeable aluminium on the Ofh level, and in the case of ANC, also with pH in KCl and the sum of alkalis BC on AE levels (Table 7). A negative correlation was also noted between the ANC values of the solutions sampled in autumn and the sum of alkaline cations, the degree of saturation with alkaline cations and the molar ratio of the sum of alkaline cations to the exchangeable aluminium on AE levels (Table 7). A positive correlation was noted between the ANC and ALK values of the solutions sampled in spring and the concentration of the exchangeable aluminium and hydrogen in Ofh levels (Table 6). These relations can be generalized by stating that the higher the pH values, the sums of alkalis (BC) and the degree of saturation with alkaline cations (V%) in the surface layers of the analyzed soils, the lower the ANC and ALK values determined in the sampled solutions, which means that there was a larger share of NO_3_^−^, Cl^−^ and SO_4_^2−^ anions than cations in the sampled soil solutions. This is probably connected with a larger rate of mineralization of organic matter and, to a smaller degree, with a decrease in the non-specific sorption of anions (mainly sulphates) in the soils fertilized with carbonate fertilizers. An increased share of sulphates in the soil solution after soil liming as a result of an increased rate of organic substance mineralization was noted by Marschner (1993) and Valeur et al. (2000, 2002). On fields fertilized with carbonate rocks, as compared to the control fields on the research plots Januszek et al. (2011), noted increased enzymatic activity as well as intensive nitrification, which allows for the statement that—on the fields fertilized with carbonate rocks—on the research plots, the rate of organic matter mineralization increased.

**Table 7:** The matrix of correlations of solution (spring/autumn) and soil properties in Ofh and AE horizons (Ofh; AE, respectively) of fields on research plots 2 years after fertilization of the whole area with ground dolomite or magnesite or serpentinite and of control fields in Wisła Forest District

Soil properties in horizons: Ofh and AE	Solution properties									
	pH	BS	ANC	ALK	Ma	Mb	Ma%	Ca/Al	Mb/Al	BC/Al
pH in H_2_O			−	−		+				
pH in KCl			−;−	−		+				
BC			−;−/−	−		+				
Exchangeable Al	+/−		+	+		−	+	;/−		
Exchangeable H^+^	/−	+	+	+		−	+			
T_e_					;+			;/−		
% Ca in T_e_										
%Mg in T_e_					/+					
V%	+/+	/+;−	−;/−	−;−		+;+	/−			
Ca^2+^:Mg^2+^										
BC:exchangeable Al	;/+	;−	−;/−	−;−		;+				
Ca^2+^:exchangeable Al										

+ positive correlation; − negative correlation; correlations determined are significant on the level *p* < 0.05; −;−/− negative correlation of the sum of alkaline cations BC on Ofh level with ANC of the solution sampled in spring and a negative correlation of soil BC on AE level from the ANC of solution sampled in spring and autumn

**Table 8:** The matrix of correlations of the properties of solutions (spring/autumn) and soil in OhA and AB horizons (OhA; AB, respectively) on the fields 2 years after fertilization of the whole area with ground dolomite or magnesite or serpentinite as well as on control fields in Ujsoły Forest District

Soil properties in horizons: Ofh and AE	Solution properties									
	pH	BS	ANC	ALK	Ma	Mb	Ma%	Ca/Al	Mb/Al	BC/Al
pH in H_2_O	/+	/+				+/+	/−			
pH in KCl	+/+	/+				+	/−			
BC	+/+		/−	/−						
Exchangeable Al						−	;/−			
Exchangeable H^+^	;+/+							−	−	−
T_e_										
% Ca in T_e_										
%Mg in T_e_		;+/+			/+		;−/−			
V%	/+	/+								
Ca^2+^:Mg^2+^					/−					
Exchangeable BC:Al					+					
Exchangeable Ca^2+^:Al										

Explanation for Table 8, see Table 7

A larger number of significant correlations between the analyzed properties of the surface soil layers and the properties of soil solutions was observed on the research plots in Wisła, whereas a smaller number was found on the plots in Ujsoły (cf. Tables 7 and 8). Exchangeable aluminium and hydrogen, the degree of saturation with alkaline cations and the molar ratio of alkaline cations to exchangeable aluminium (BC:Al) in the sorptive complex of the examined surface layers were correlated with the properties of soil solutions in the case of the soils in Wisła. On the research plot in Ujsoły, there was a positive correlation between the pH values in H_2_O as well as in KCl on OhA levels and the pH values as well as the degree of saturation with alkaline cations (BS) in solutions sampled in autumn (Table 8). A positive correlation was also noted between the pH values in KCl on OhA levels and the pH values of solutions sampled in spring (Table 8). A positive correlation was found between the pH values in H_2_O as well as in KCl in OhA horizons and the sum of alkaline cations in solutions sampled in spring, and a negative correlation was found between the pH values in H_2_O as well as in KCl in OhA horizons and the level of acidity (Ma%) of solutions sampled in autumn (Table 8). A positive correlation was noted between the share of magnesium in the soil sorptive complex in ABbr horizon (10–20 cm) and the degree of saturation of solutions sampled both in spring and in autumn with alkaline cations; a negative correlation was noted between the share of magnesium in the soil sorptive complex in ABbr horizon (10–20 cm) and the degree of acidity of soil solutions sampled both in spring and in autumn (Table 8). As on the research plot in Wisła, also in Ujsoły, a negative correlation was found between the sum of alkaline cations in Ofh and OhA horizons and values of ANC and ALK of solutions sampled in autumn, which means an increase in anions, NO_3_^−^, Cl^−^ and SO_4_^2−^, in solutions sampled from soils characterized by a higher pH of the fertilized fields (Table 7).

## Discussion

Norway spruce is one of the most common and economically important tree species in Europe. Sustainable management of spruce forests in a changing environment presents an enormous challenge for European forestry. Knowledge about forest growth reactions and growth trends is just one important aspect of sustainable forest ecosystem management (Spiecker 2000). The growth of stands may be strongly influenced by soil preparation, selection of species and provenances. Fertilizers and lime have been applied to some European forests for many decades in order to increase site productivity and to overcome some effects of site degradation caused by former land use. Kulhavý (2000) evaluated the simulated input of sulphur—in situ—with the parallel application of dolomitic limestone. Soil pH increased significantly in the surface humus within the course of 5 years but was insignificant in the mineral soil. Liming resulted in the decrease of leaching of humic acids and improvement of soil saturation with base cations. Soil solution showed lower acidity, higher conductivity and higher content of Ca and Mg. From 2003, the Norway spruce decline started in the Beskid Śląski and Żywiecki (western edge of the Carpathians). Kulhavý (2000) was the first to investigate this nature. By comparison, our results indicate a positive change of soil properties—liming resulted in the decrease of leaching of humic acids, and improvement of soil saturation with basic cations and soil solution showed lower acidity, higher conductivity and higher content of Ca and Mg.

Liming forest soils causes beneficial effects such as reduced acidity, reducing the concentration of toxic forms of aluminium and the increase in the supply of Ca and Mg. The liming causing other side effects in some site conditions (intensity of nitrification and the threat of surface water with nitrates, too fast mineralization and loss of organic matter, increased CO_2_ emissions, shortness of spruce root systems, boron deficiency, increased activity of root pathogens, increased trunks with butt-end rot, fall growth stands) (Kreutzer 1995). A higher concentration of exchangeable hydrogen in the sorption complex in the mineral horizons of the examined soils may be one of the causes of growth (increment) inhibition in spruce stands after liming, which was concluded following experiments conducted in the Nordic countries (Ingerslev 1997; Sikström 1997), as well as root system shallowing noted for spruce after liming (Kreutzer 1995). For this reason, Małek (2009) planted seedlings 2 years after fertilization to avoid exposing the young seedlings to stress from increased acidification of the soil solutions in the upper mineral horizons. It is conceivable that the concentration of hydrogen and aluminium in the soil solutions in the upper mineral horizons in the following years will increase (Guckland et al. 2012), which may contribute to the inhibition of growth and development of new seedlings as well as stands which grow there.

The antagonism between calcium and magnesium can decide about the negative impact of liming on plant growth. It is supposed that a harmful effect of high doses of lime on the yield of plants is caused by an imbalance between calcium and magnesium in the soil (Gorlach & Gorlach 1983a). The correct ratio of Ca:Mg in the soil and in the plant may be an important factor in optimal plant growth. The correct ratio of these macronutrients depends on the plant species. According to Warchołowa (Gorlach & Gorlach 1983a), the ratio Ca:Mg = 1:1 is the most favourable for plants requiring low calcium levels (e.g. grasses). The physiological needs regarding calcium, particularly in coniferous trees, are relatively small (Huber et al. 2004). The pot experiments carried out by Gorlach & Gorlach (1983a, b) show that MgCO_3_ used in a dose according to the 0.5 and 1.0 hydrolytic acidity (Hh) worked depending on the species and variety of plant, similarly or better than CaCO_3_, but MgCO_3_ used in a dose 2.0 of Hh with the exception of some plants used in the experiment, significantly inhibited the growth of plants. The negative impact on the yield of MgCO_3_ plants depended on soil properties and decreased with increasing sorption capacity of the soil. It was associated with a significant decrease in Ca content in plants and the reduction of the equivalence ratio Ca:Mg in aboveground parts to less than 1 (Gorlach & Gorlach 1983a). Magnesium fertilization was negative that liming affected B, Cu, Mn and Zn intake from plants, while it positively affected the intake of Mo (Gorlach & Gorlach 1983b). According to research conducted by Silva et al. (2001a, 2001b), magnesium was 100 times more effective than calcium in relieving aluminium toxicity for roots. Dolomitic soil spruce on the experimental plot in Höglwald (southern Bavaria) had only a significant and permanent effect on the concentration of Ca in the needles. Magnesium concentration did not increase in the same way, despite the fact that the amount of Ca and Mg in the dolomite was the same (Huber et al. 2004). The physiological need for calcium, especially in conifers, is relatively small. Calcium uptake by plant roots is mainly a passive process because plants cannot avoid absorbing Ca in excess. Hence, Ca must change in the form of detoxification of calcium oxalate (Huber et al. 2004). As a result of fertilization, ratio of Ca:Mg was significantly reduced in the soils and soil solutions. This has a positive impact on facilitating the intake of magnesium by the stands and plantings under the canopy of weakened forests. The effects of liming (2.0 and 4.0 Mg ha^−1^) on chemical properties of soil, nutrient concentrations of needles and growth of Scots pine (*Pinus sylvestris* L.) transplants were investigated by Saarsalmi et al. (2011). The effect of liming is visible mainly in the humus layer and in the upper mineral layer A (Šrámek et al. 2012). In the deeper mineral soil (down to 30 cm), only the increase of pH and exchangeable magnesium was found significant. Effects of Mg fertilization on yellowing of Norway spruce needles at higher elevations of the Šumava Mountains, Czech Republic were investigated by Vacek et al. (2006). The fertilization resulted in stabile foliation while marked defoliation was reported from control plots in both vegetation zones. Magnesium deficiency can be effectively eliminated through fertilization. Balanced nutrition contributes to long-term vigour and stability of forest stands.

A significant increase in the concentration of nickel in the Ofh horizon of soil on the Wisła plots fertilized with serpentinite did not contribute to a reduction in enzyme activity, which will be the subject of a separate publication.

## Conclusions

The liming of forest soils, characterized by thick, strongly acidified surface organic layers, by using single, large doses of carbonate fertilizers, contributes to an increase in the concentration of hydrogen in surface mineral levels. The litter was released from organic colloids of the higher located organic levels; this phenomenon may contribute to reductions in stand growth rates. Forest soil liming is recommended with the use of low doses of carbonate calcium and magnesium fertilizers, which make these layers reach pH in 1 M KCl that does not exceed the value of 4.5.

To enrich acidified forest soils with surface humus layers with alkaline cations and to avoid stronger acidification of lower mineral levels after liming, it is safer to use slow-performance silicate fertilizers. We recommend those containing calcium and/or magnesium and/or potassium, depending on the range of the deficit alkaline cations.

As a result of magnesite and serpentinite fertilization, ratio of Ca:Mg was significantly reduced in the soils and soil solutions. This has a positive impact on facilitating the intake of magnesium by the stands and plantings under the canopy of weakened forests.

Differences in the composition and concentrations of the analytes in waters from these research plots, related to the periods of their sampling, are connected with the chemical composition of the fertilizers applied and rate of mineralization. This indicates further progress of the processes of removal of Ca^2+^ by Mg^2+^ as well as increased removal of Al and H from the sorptive complex.

The saturation of the analyzed waters with alkalis (BS) was very low, especially in Wisła Forest District .The values obtained show moderate flexibility of soil water solutions in relation to the acid load, whereas the results from the plots in Ujsoły Forest District indicate high flexibility of the solutions. The opposite tendencies were noted for the degree of soil acidity (Ma%). The fertilization applied did not basically change these values. The acid-neutralizing capacity (ANC_aq_) of the analyzed waters increased on research plots as did alkalinity. These waters may respond with strong pH fluctuations even to the smallest changes in the composition of the solution.

There was further change of the reaction of soil waters and of the saturation with alkalis (BS) with simultaneous lowering of the degree of acidity (Ma%), especially on plots with dolomite and, above all, in Wisła Forest District. The phenomenon intensified during the growing season. Fertilization with magnesite increased the saturation of the solid soil phase with the alkalis of the exchangeable complex (Mb), mainly in Wisła Forest District. In this forest district, soils undergo leaching of metal hydroxides (aluminium buffer), whereas in Ujsoły Forest District, alkaline cations were released from silicates and exchangers. The saturation of soils water under spruce stands in Wisła Forest District with alkalis is moderate; it is high in Ujsoły Forest District.

A negative correlation was noted between the pH values, the sum of alkalis as well as the degree of saturation of the sorptive complex with alkalis in surface horizons of the analyzed soils and the acid-neutralizing capacity as well as alkalinity of the analyzed solutions at the depth of 20 cm. This relationship may be explained by an increased rate of organic matter mineralization and the activation of the nitrification process.
